# LdEP01, the first characterized *Lycorma delicatula* salivary effector protein modulates plant defenses

**DOI:** 10.1007/s11033-025-11288-3

**Published:** 2025-12-11

**Authors:** Alexandrea G. Smith, Julien G. Levy, Flor E. Acevedo, Cecilia Tamborindeguy

**Affiliations:** 1 Department of Entomology, Texas A&M University, College Station, TX 77843 USA; 2 Department of Horticultural Sciences, Texas A&M University, College Station, TX 77843 USA; 3 Department of Entomology, Penn State University, University Park, PA 16802 USA

**Keywords:** Spotted lanternfly, Salivary protein, Calcium signaling, Herbivore offense, Hemiptera

## Abstract

The spotted lanternfly (SLF), *Lycorma delicatula*, is a polyphagous invasive species in the United States known for causing significant damage to grapevines. This phloem-feeding insect secretes saliva containing effector proteins at the time of feeding, allowing for the ingestion of the phloem’s nutrients. The mechanisms that SLF uses to modulate plant defenses and feed efficiently remain unknown and understudied. Methods and Results: We identified LdEP01, an SLF predicted secreted effector protein with a FK506 binding protein (FKBP)-type domain and two EF-hand Ca^2+^ binding domains. LdEP01 is highly expressed in the salivary glands compared to the SLF’s full body. LdEP01 modulates plant defenses by suppressing cytosolic calcium accumulation and suppressing hypersensitive response (HR). Functional analysis confirmed that LdEP01 binds to calcium but does not undergo conformational change when bound to calcium. Conclusions: These findings suggest that LdEP01 plays a crucial role in modulating plant defenses, allowing SLF to feed efficiently on plants.

**Supplementary Information:**

The online version contains supplementary material available at 10.1007/s11033-025-11288-3.

## Introduction

The spotted lanternfly (SLF), *Lycorma delicatula* (Hemiptera: Fulgoridae), is a phloem-feeding insect. This devastating invasive pest arrived in the Northeastern United States in 2014 [1] causing important losses to the grapevine and other tree fruit industries [2–4]. While strict quarantines were put in place in the affected region, this insect continues to spread, increasing its geographical range and threatening crops [5]. The spotted lanternfly feeds by piercing the plants with its stylet and sucking nutrients from the phloem, consuming large amounts of phloem sap. Further, it also produces large quantities of honeydew, which promotes the development of sooty mold [6]. When feeding, phloem-feeding insects secrete two types of saliva (gelling and watery). The “gelling” saliva aids the insect during feeding by forming a sheath that protects the stylet [7]. The “watery” saliva is secreted during probing of the cells encountered along the stylet path until it reaches the phloem, and also during active feeding from the sieve elements. Probing allows the insect to evaluate the suitability of the plant as a host and to determine when the phloem has been reached [7].

Hemipteran phloem-feeding insects rely on salivary effector proteins secreted during feeding in the gelling or watery saliva to feed efficiently [8]. These effector proteins play different roles, such as modifying the phloem composition or suppressing plant defenses to feed with minimal detection from the host plant [9]. Indeed, several hemipteran salivary effector proteins have been identified as interfering with plant defenses, such as modulating the accumulation of reactive oxygen species (ROS) and calcium concentrations, both of which act as signaling molecules [10–12], or suppressing or inducing a hypersensitive response (HR) [13, 14]. An influx of ROS into the cell, known as an oxidative burst, acts as a signal to induce defenses locally and systemically. On the other hand, a large accumulation of ROS also acts as a defense mechanism and can lead to HR at the site of herbivory or pathogen threat [15]. HR is a localized programmed cell death at the point of attack, which can stop the spread of pathogens or deter insect feeding [16]. Similarly, calcium is another signaling molecule plants use. Upon attack, a large influx of calcium from the apoplast and organelles into the cytoplasm elicits a variety of responses. In the sieve elements, calcium bursts lead to phloem plugging by the deployment of P-proteins and deposition of callose at the sieve plate; these events disrupt phloem flow and deter feeding [17]. Therefore, disrupting calcium signaling is essential for the success of phloem-feeding insects. Indeed, calcium-binding effectors secreted during feeding and interfering with plant defenses have been characterized [12]. Since phloem-feeders are major vectors of plant pathogens, the manipulation of plant defenses by effector proteins not only enables the insect to feed efficiently causing a decline in plant yield, it can also mediate its vector capacity [18].

Salivary effectors have been studied in several hemipteran species; overall, it appears that many of those are unique to specific insect families, while others are found in many species [8]. This study contributes to the identification of the mechanisms used by the invasive spotted lanternfly to feed on plants and to a further understanding of phloem-feeding hemipteran interaction with plants. Additionally, this study also contributes to the field of plant immune responses and is the basis for determining resistance genes to control this invasive and devastating pest. Here, we identified the effector protein LdEP01, the first spotted lanternfly salivary effector, and characterized its role in manipulating plant defenses.

## Materials and methods

### Bioinformatic analyses to identify LdEP01 as a candidate salivary effector

LdEP01 was identified by mining transcriptomic data from adult *L. delicatula* salivary glands (transcriptome data is in preparation for publication). Specifically, we selected candidate proteins predicted to encode a signal peptide using SignalP 6.0 to identify proteins targeted for secretion by the sec secretion system [19]. Then, the potential extracellular localization of the candidate effector was determined using WoLF PSORT (https://wolfpsort.hgc.jp/). To identify a putative function for the identified candidate, blast search was conducted against the nr database. Finally, specific domains were predicted using InterProScan [20].

### RNA extraction and cDNA synthesis

Spotted lanternfly adults were collected from the field in the SLF quarantine zone of Pennsylvania, United States. Immediately following collection, salivary glands were dissected and preserved in RNAlater (Thermo Fisher Scientific, Waltham, MA) at −80 °C until used for RNA extraction. RNA was extracted from salivary glands or whole bodies using the Trizol RNA extraction method (Thermo Fisher Scientific). One mg of RNA was reverse transcribed using the Verso cDNA Synthesis Kit (Thermo Fisher Scientific).

### Gene expression analysis by qPCR

For qPCR analysis, reactions included 10 ng of cDNA, 250 nM of each primer (Table S1), and 5 µL of PowerUp SYBR Green Master Mix (Applied Biosystems, Waltham, MA); nuclease-free water was used to adjust the volume to 10 µL. The amplification protocol consisted of a 2-minute incubation at 95 °C followed by 40 cycles of 95 °C for 15 s and 60 °C for 30 s using the QuantStudio 6 Flex Real-Time PCR machine (Thermo Fisher Scientific). Three technical replicates were completed for each tissue type and a negative control (no cDNA) in each replicate. The average threshold cycle (Ct) values and standard error were calculated. The SLF gene, 18 s ribosomal RNA gene (JX556762.1) was used as the reference. ΔCt was calculated from the average of three biological replicates. Statistical analyses were conducted using the two-tailed t-test to compare gene expression levels between the SLF full body and salivary glands in R (https://www.r-project.org/).

### Cloning

The candidate effector LdEP01 was amplified from the cDNA for cloning (GenBank; PV590600). Primers were designed to amplify the full-length coding sequence as well as the sequence coding for the mature protein, i.e., minus the signal peptide. PrimeStar GXL DNA polymerase (Takara Bio, San Jose, CA) was used for PCR amplification. The reactions included 10 mL of 5X PrimeSTAR GXL Buffer, 4 mL of dNTP (2.5 mM each), 0.2 mM of each primer (Table S1), and 1 mL of PrimeSTAR GXL DNA Polymerase. Water was added to bring the volume to 50 mL. The PCR was performed using a thermocycler (Applied Biosystems) with 98 °C for 3 min; 40 cycles of 98 °C for 10 s, 55 °C for 15 s, and 68 °C for 1 min; and 68 °C for 10 min. The obtained amplicons were verified by sequencing and cloned into pDONR-201 using BP Clonase II (Thermo Fisher Scientific) and pEarleyGate101 (pEG101) plasmid by LR Clonase II following the manufacturer’s protocols. The pEG101 vector was used for cloning; this vector includes an EYFP and HA tag. The full-length and mature candidate effector protein sequences were cloned with EYFP and HA tags in frame at their C-terminus, allowing detection of the expressed proteins. The full-length LdEP01 sequence was also cloned into Champion™ pET101 (Thermo Fisher Scientific) for protein expression in *Escherichia coli*. All constructs were verified by sequencing.

### Agrobacterium transformation and agroinfiltration

The pEG101 recombinant vectors with the gene of interest corresponding to the full-length and mature proteins were transformed by electroporation into the GV3101 strain of *Agrobacterium tumefaciens*. Bacterial cultures were grown in 5 ml of LB broth with kanamycin (50 mg/ml), 10 mM morpholineethanesulfonic acid (MES), and 100 µM acetosyringone overnight at 28 °C with shaking to prepare for infiltrations. Following the overnight culture growth, the bacterial cells were pelleted by centrifugation, resuspended in infiltration buffer, and left shaking in the dark for at least 2 h. The infiltration buffer contained 200 µM acetosyringone, 10 mM magnesium chloride, and 10 mM MES. Leaves of 4- to 6-week-old *Nicotiana benthamiana* plants were infiltrated with syringes without needles. After infiltration, the plants were left on shelves at room temperature (23 to 26 °C) for the time needed for the experiments [21].

### Candidate effector expression verification in N. benthamiana leaves by localization and Western blot analysis.

Forty-eight to 72 h after agroinfiltration of the genes of interest cloned in the pEG101 vector, the same leaf areas were infiltrated with 4’,6-diamidino-2-phenylindole (DAPI) to stain cell nuclei. EYFP and DAPI signals were detected using a fluorescent Revolution microscope (ECHO, San Diego, CA) with the FITC (470 nm, green) and DAPI (380 nm, blue) filters (Fig. S1).

Also, we homogenized infiltrated leaf tissues and extracted proteins using a protein lysis buffer (10 µM Tris-HCL, 0.5 µM EDTA, 0.4 µM DTT, 1X Protease inhibitor cocktail (Thermo Fisher Scientific). The purified proteins were loaded with 4× LDS sample buffer onto a 4 to 12% Bis-Tris NuPage gel (Thermo Fisher Scientific) and separated by electrophoresis at 120 V. The proteins were then transferred from the Bis-Tris gel to an Immobilon-P PVDF membrane (MilliporeSigma, Burlington, MA) using a wet overnight transfer system. The membrane was then blocked with 5% dry milk resuspended in TBST (20 mM Tris-HCl, pH 7.6, 150 mM NaCl, 0.1% Tween-20) buffer, followed by incubation with polyclonal anti-HA HRP-conjugated primary antibody (Thermo Fisher Scientific) diluted (1:500) in TBST with 5% dry milk at room temperature for 2 h. The membrane was then washed with TBST. The bound antibody was detected using the SuperSignal™ West Pico PLUS Chemiluminescent Substrate (Thermo Fisher Scientific) and imaged using the iBright 1500 imaging system (Thermo Fisher Scientific) (Fig. S2). These tests were conducted to validate the expression of the protein of interest in *N. benthamiana*.

### Ca^2+^ assay

Agrobacterium cultures at 0.7 OD^600^ were prepared and infiltrated into SLJR15 transgenic *N. benthamiana* leaves as previously described. The SLJR15 transgenic line expresses the reporter protein aequorin [22, 23]. Leaf disks were punched out from the infiltrated areas 24 h after infiltration. The leaf disks were placed individually in 100 ml Reconstitution Buffer (RB) with 1 mM coelenterazine overnight, with the adaxial face down in the wells of a flat-bottom white 96-well plate (Greiner Bio-One, Monroe, NC). The RB consisted of 2 mM MES and 10 mM CaCl_2_. The following day, the RB with coelenterazine buffer was removed from the plate, and the leaf discs were washed with RB twice. Following the washes, 50 ml of RB was added to each well. The luminescence was measured using a Tecan NanoQuant plate reader (Morrisville, NC) for 5 cycles; then, 50 ml of RB with 1 µM flg22 (GeneScript) or 0.1 M chitin (Thermo Fisher Scientific) were added to each well, and the luminescence was measured for 35 additional cycles. This assay was repeated at least 3 times; in each independent repetition, each candidate was agroinfiltrated into 3 different plants (biological replicates). Four leaf discs were collected from each infiltrated area and used as technical replicates. Agroinfiltration with the empty vector was used as a control. We verified that a calcium burst was not induced when the elicitor was not included (Fig. S3).

### ROS assay

ROS assays were performed as described Levy, et al. [24]. Briefly, wild-type *N. benthamiana* leaves were agroinfiltrated as previously described. Twenty-four hours after the infiltration, disks of the infiltrated leaves were collected. The leaf disks were placed individually with the adaxial face down in a flat-bottom white 96-well plate (Greiner Bio-One) and incubated in H_2_O overnight. The following day, the water was removed, and after washing the leaf disks with water, 50 ml of buffer consisting of 1 µM horseradish peroxidase (Thermo Fisher Scientific) and 85 mM luminol (Ward’s Science, Rochester, NY) were added to each well. The luminescence was then measured using the Tecan NanoQuant plate reader for 5 cycles; then 50 ml of buffer with 1 µM flg22 or 0.1 M of chitin were added to each well, and the luminescence was measured for 35 additional cycles. This assay was repeated at least 3 times; in each independent repetition, each candidate was agroinfiltrated into 3 different plants. Four leaf discs were collected from each infiltrated area and used as technical replicates. The experiment included infiltrations with *A. tumefaciens* carrying an empty vector as a control. We verified that ROS did not accumulate when the elicitor was not included (Fig. S3).

### Cell death assay

*Nicotiana benthamiana* leaves were infiltrated as previously described. The genes of interest were agroinfiltrated into *N. benthamiana* at 0.7 OD^600^. To evaluate whether the genes of interest induced cell death, HR was scored 10 days after agroinfiltration. To evaluate whether they could disrupt cell death, the genes of interest were co-infiltrated with Prf^D1416V^ at 0.3 OD^600^, a gene that induces cell death in *N. benthamiana* [25]. HR was scored 7 days after agroinfiltration. As controls, Prf^D1416V^ was co-infiltrated with an empty pEG101 vector,, and with AvrPto1, a gene that suppresses Prf^D1416V^-induced cell death [26]. For HR scoring, a 0–10 scale where 0 was considered no cell death (no HR) and 10 represented cell death of the full infiltrated area was used for both single and co-infiltrations. These assays were repeated at least 3 times with each construct. In each repetition, at least 3 plants were infiltrated, and at least three leaves per plant were infiltrated with each construct.

*LdEP01 Protein Expression and Purification in BL21* *E. coli*.

The LdEP01 full-length sequence cloned into pET101 vector was transformed into BL21 (DE3) *E. coli* cells. A single *E. coli* colony was cultured overnight in LB containing ampicillin at 37 °C. The next day, expression of the LdEP01 protein was induced with 0.5 mM IPTG following the manufacturer’s instructions. Six hours later, the cells were pelleted, and proteins were extracted from the pellets using B-PER reagent (Thermo Fisher Scientific). The LdEP01 protein was purified using a His60 Ni Gravity Column (Takara). Briefly, the LdEP01 protein was bound to the column using an automatic tube rotator at 4 °C for 2 h. The column was washed with 10 column volumes of equilibration buffer (50 mM sodium phosphate, 300 mM sodium chloride, 20 mM imidazole; pH 7.4) followed by 10 column volumes of wash buffer (50 mM sodium phosphate, 300 mM sodium chloride, 40 mM imidazole; pH 7.4). The sample was then eluted from the column with an elution buffer (50 mM sodium phosphate, 300 mM sodium chloride, 300 mM imidazole; pH 7.4). The imidazole was removed from the purified protein product using Amicon Ultra-0.5 ml centrifugal filters (MilliporeSigma). The expression and purification of the protein were confirmed by SDS-PAGE with SimplyBlue SafeStain (Fig. S4A) (Thermo Fisher Scientific) and western blot using 6x-His Tag Monoclonal Antibody (Thermo Fisher Scientific) as previously described (Fig. S4B).

### Ruthenium red staining of LdEP01

The purified target protein was used for ruthenium red staining following the protocol in Anisuzzaman, et al. [27]. The LdEP01 protein was subjected to SDS-PAGE under heating-reducing conditions. BSA was also loaded into the gel and used as a negative control for this experiment; ruthenium red binds to calcium-binding proteins and should not bind to BSA. The proteins were then transferred to a PDVF membrane overnight at 30 V. Once transferred, the membrane was washed with ruthenium red buffer (51.4 mM KCl, 5.04 mM MgCl, 10 mM Tris-HCl pH 7.5) for 15 min. Once washed, the membrane was stained with ruthenium red stain (25 mg/L dissolved in ruthenium red buffer) for 15 min.

### Mobility shift assay for candidate LdEP01

The purified LdEP01 protein was used for a mobility shift assay following the protocol described in Ye et al., 2017 [12]. The purified protein was incubated for 30 min at 25 °C with 0.5 or 3 mM CaCl2 or 0.5 mM EDTA (final concentrations in the sample). The samples were then mixed with 2x Laemmli buffer (Bio-Rad) and then subjected to native protein gel electrophoresis in non-heating conditions and stained with SimplyBlue SafeStain (Thermo Fisher Scientific). PageRuler™ Plus Prestained Protein Ladder (Thermo Fisher Scientific) was used as a reference for protein size.

### Statistical analyses

For the ROS and calcium assays, the results from the technical replicates were averaged. The Wilcoxon rank-sum test was performed to compare the luminescence levels obtained between the signal from the empty vector and the LdEP01 gene with or without the signal peptide. For the HR, Wilcoxon rank-sum test was conducted to compare the scores of co-infiltrations of candidates with Prf ^D1416V^ and the co-infiltration Prf ^D1416V^-EV. All analyses were performed with R, version 2024.12.0 + 467 (https://www.r-project.org/).

## Results

### LdEP01 protein description

**Fig. 1:**
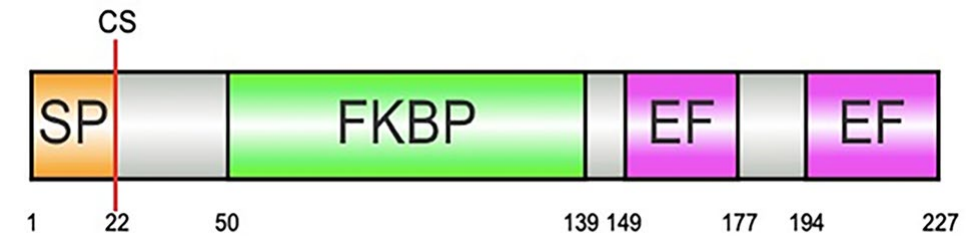
Protein structure of LdEP01 based on SignalP, InterProScan, and PROSITE analyses. SP: signal peptide (1–22 aa); CS: cleavage site (cleaved between 22–23 aa); FKBP: predicted domain FKBP-type (50–139 aa); EF: two EF-hand calcium binding domains were predicted (149–177 and 194–227 aa)

The LdEP01 sequence from the spotted lanternfly salivary gland transcriptome was identified as a putative salivary effector. The predicted protein was 227 amino acids long. SignalP identified a 22-amino acid-long signal peptide with a probability of cleavage greater than 0.97. The protein was predicted to have an extracellular localization using WoLF PSORT [28]. Domain prediction using InterProScan and PROSITE identified an FKBP-type domain (PS50059) and two EF-hand calcium binding domains (PS00018) (Fig. 1). The best hit identified by blast analysis was the *Nilaparvata lugens* peptidyl-prolyl cis-trans isomerase FKBP14 (XP_039286805) with 79% identity.

### LdEP01 expression analysis

**Fig. 2:**
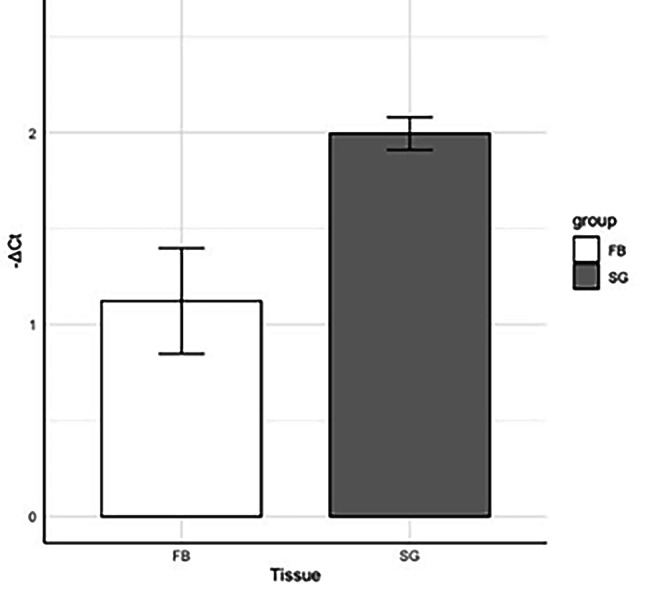
RT-qPCR analysis of gene expression in full body (FB) and salivary gland (SG) tissues. The bar graph shows the average ΔCt values with standard error of the mean for FB and SG tissues using 18 S ribosomal RNA as the reference gene. The relative expression was compared using Student’s *t-*test (*P* ≤ 0.05). Significant *p-*values are indicated with asterisks. (*): *p* ≤ 0.05. LdEP01 had a higher relative expression in the salivary gland tissues compared to the full body

The expression of LdEP01 was evaluated using RT-qPCR in full body and salivary glands from the spotted lanternfly. Higher relative gene expression was detected in the salivary glands of the spotted lanternfly than the full body. A significant difference in gene expression was determined using Student’s t-test (*P* ≤ 0.05) t = −3.0284, df = 4, p-value = 0.03884 (Fig. 2).

### Calcium burst is suppressed by the spotted lanternfly effector LdEP01

**Fig. 3:**
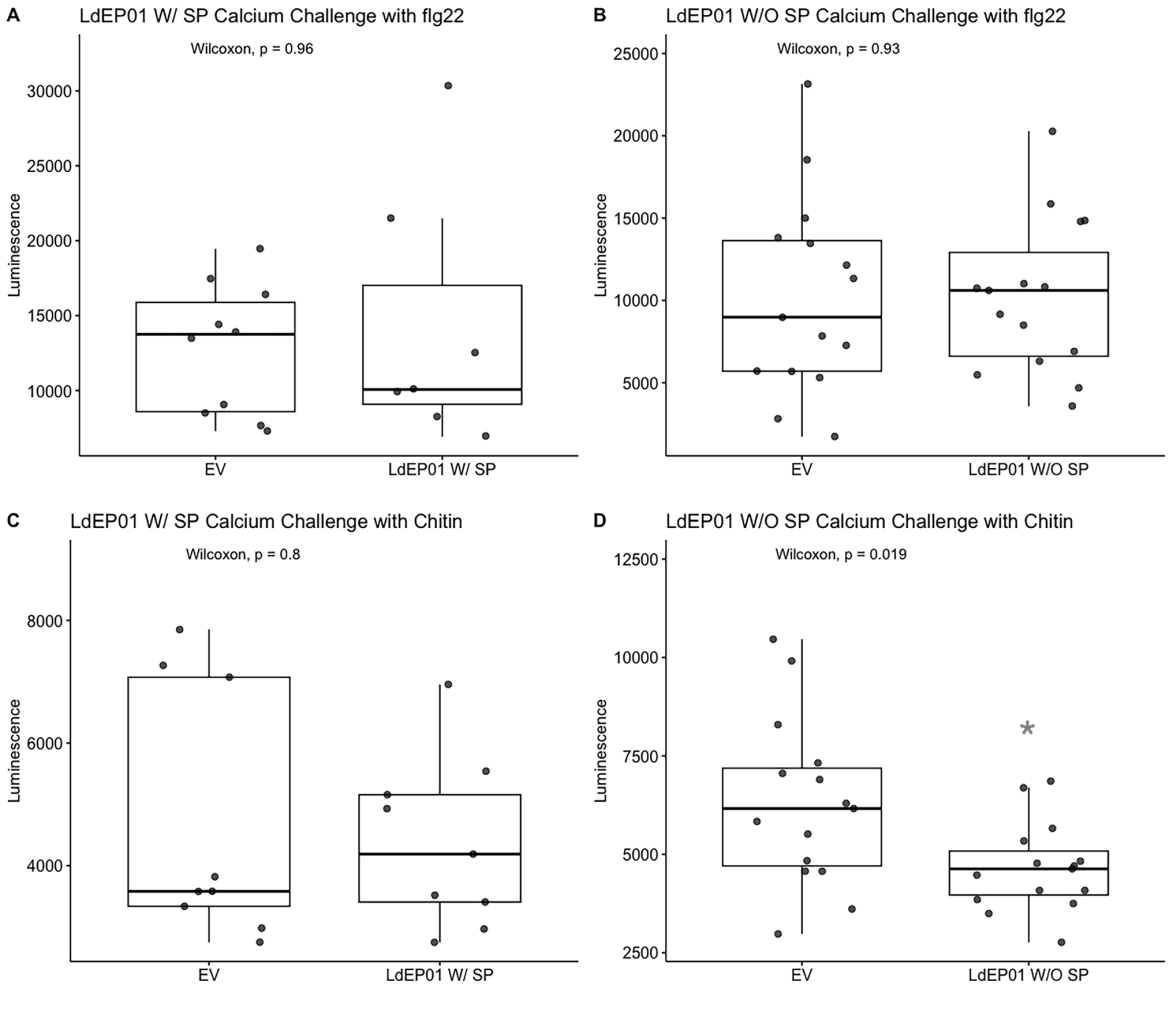
Calcium accumulation assays. The luminescence signals corresponding to cytoplasmic calcium accumulation were measured after flg22 and chitin challenge. The Y-axis reports the accumulated luminescence measured. The signals obtained for leaf disks agroinfiltrated with the genes of interest were compared against empty pEG101 (EV) vector using the Wilcoxon rank-sum test. Significant *p-*values are indicated with asterisks. (*): *p* ≤ 0.05. **A** and **B** show the results for the full-length (LdEP01 W/SP) and mature (LdEP01 W/O SP) proteins challenged with flg22, respectively. **C** and **D** report calcium accumulation following chitin challenge

Agrobacterium strains carrying constructs encoding the full-length and mature proteins were infiltrated into SLJR15 transgenic *N. benthamiana* to measure cytosolic calcium levels after flg22 or chitin challenge. LdEP01 protein with or without the signal peptide did not modulate calcium levels after flg22 challenge when compared to leaves agroinfiltrated with an empty pEG101 vector (negative control) (Fig. 3A and B). When challenged with chitin, only LdEP01 without the signal peptide (W/O SP) was able to modulate and suppress cytoplasmic calcium levels (Fig. 3D).

### ROS signaling is not suppressed by the spotted lanternfly effector LdEP01

**Fig. 4:**
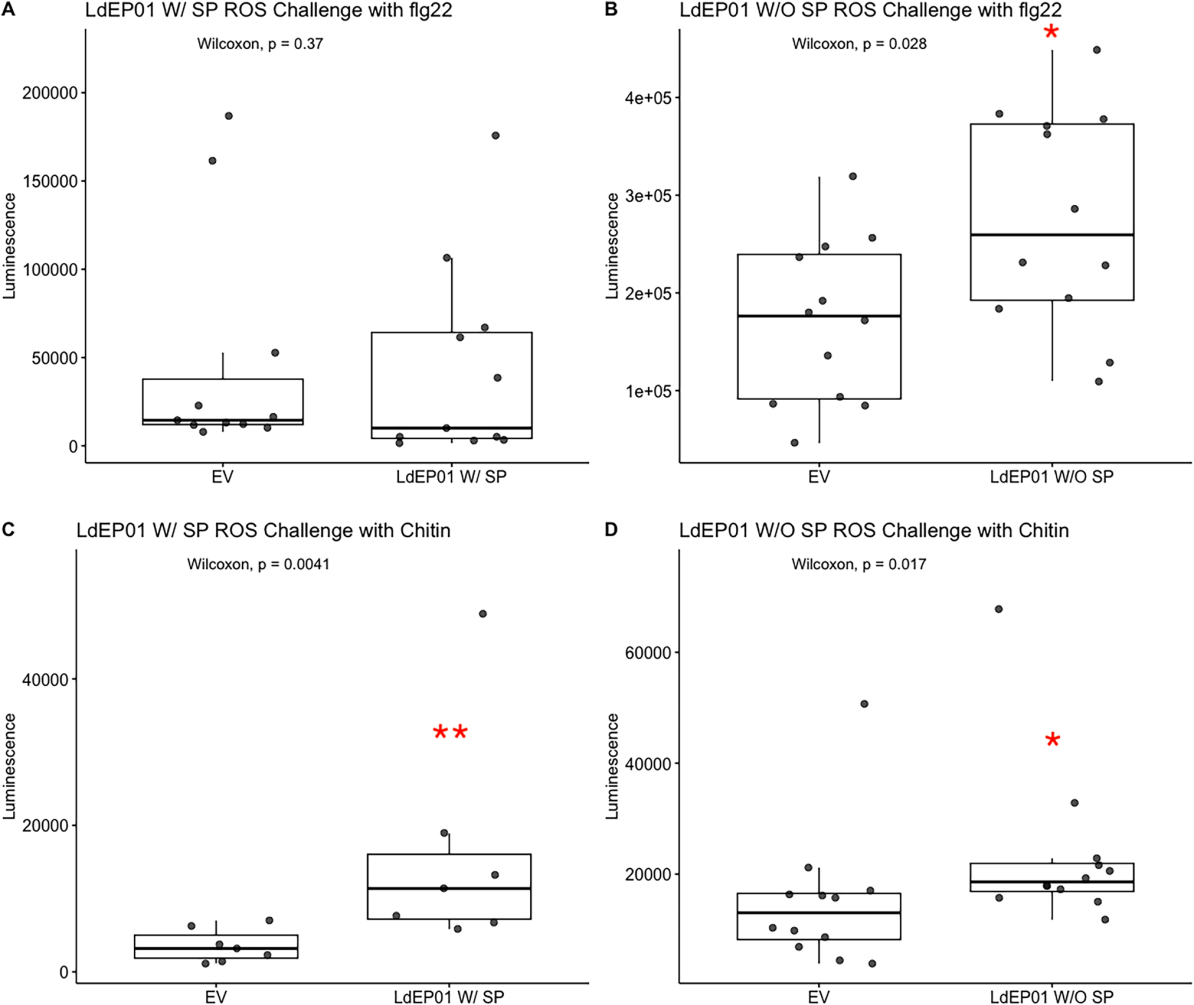
ROS accumulation assay. ROS levels were measured in *N. benthamiana* leaf disks expressing each construct after flg22 or chitin challenge. The Y-axis reports the accumulated luminescence measured. The luminescence produced by the leaf disks expressing the genes of interest was compared to that of the leaf disc agroinfiltrated with the empty pEG101 vector (EV) using Wilcoxon rank-sum test. Significant p-values are indicated with asterisks (*): *p* ≤ 0.05; (**): *p* ≤ 0.01. **A** and **B** show the results for the full-length (LdEP01 W/SP) and mature (LdEP01 W/O SP) proteins challenged with flg22, respectively. **C** and **D** report ROS accumulation following chitin challenge

Agrobacterium strains carrying the LdEP01 gene with or without the signal peptide were infiltrated into wild-type *N. benthamiana* leaves for ROS assays. Except for the construct expressing the full-length LdEP01 challenged with flg22 (Fig. 4A), all other assays resulted in significantly higher ROS accumulation in the challenged leaf disks expressing LdEP01 compared with disks expressing the empty vector (Fig. 4B and C, & 4D).

### Hypersensitive response is suppressed by the spotted lanternfly effector LdEP01

**Fig. 5:**
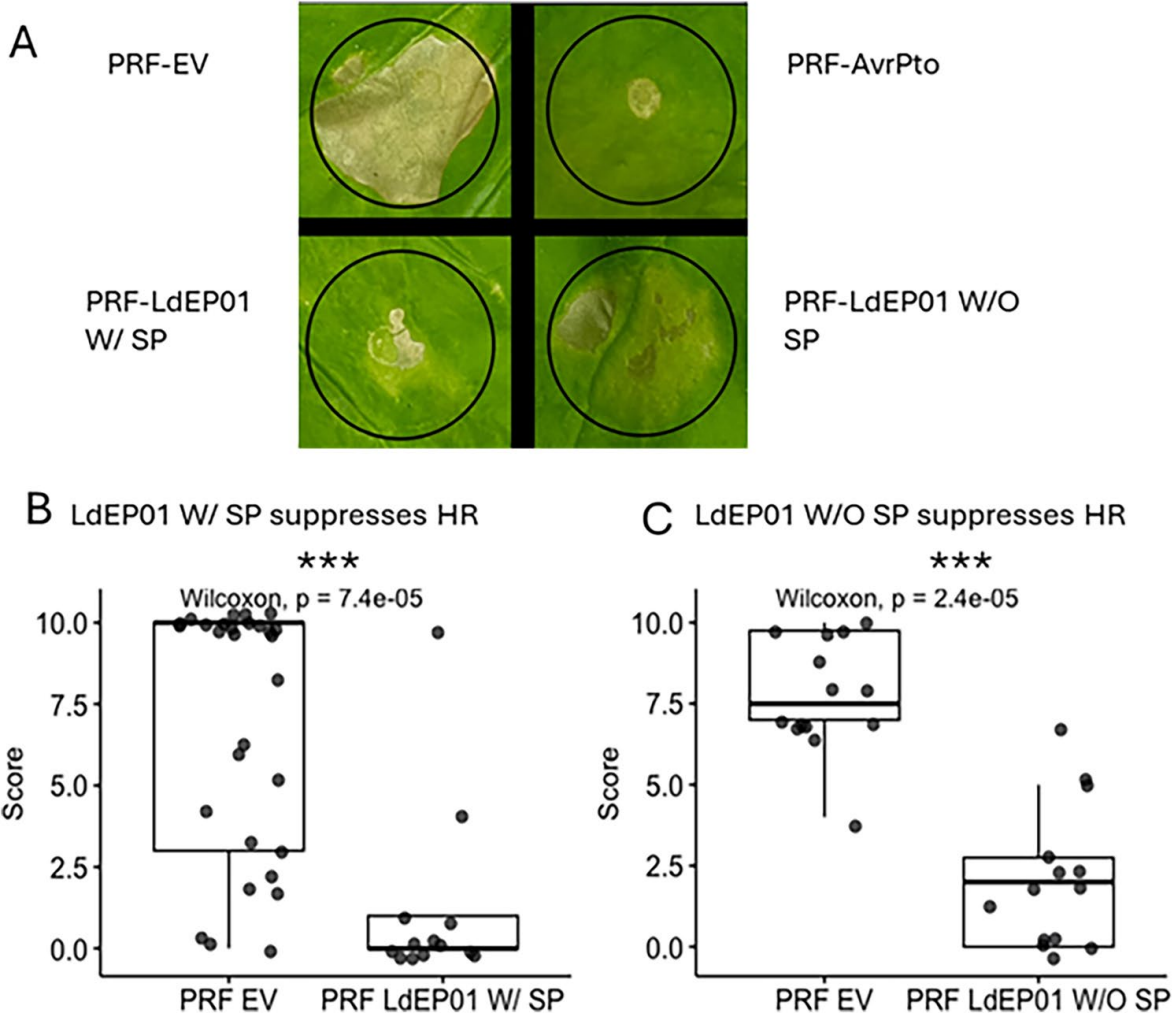
Hypersensitive response suppression assay. **A** Agroinfiltrated *N. benthamiana* leaves with positive control and negative controls, along with genes of interest co-infiltrated with Prf^D1416V^. Circles indicate the area that was infiltrated. **B** Boxplot displaying the cell death scores on a scale from 0–10 for the co-infiltrations of Prf^D1416V^ with the empty vector and with LdEP01 with the signal peptide. **C** Boxplot displaying the cell death scores on a scale from 0–10 for the co-infiltrations of Prf^D1416V^ with the empty vector and with LdEP01 without the signal peptide. The score obtained for the co-infiltration of the constructs of interest with Prf^D1416V^ was compared to the co-infiltration of Prf^D1416V^ and an empty vector (EV) using the Wilcoxon rank-sum test. Significant p-values are indicated with asterisks. (***): *p* ≤ 0.001

The candidate effector LdEP01 was cloned with and without the signal peptide into the pEG101 vector and agroinfiltrated into *N. benthamiana* to test for the induction of HR. Neither construct induced significant cell death (not shown). The candidate constructs were also co-infiltrated with Prf ^D1416V^, a known HR inducer in *N. benthamiana*, to evaluate if they could reduce Prf ^D1416V^-induced cell death. Both LdEP01 constructs efficiently suppressed cell death when co-infiltrated with Prf ^D1416V^ (Fig. 5).

### LdEP01 binds to Ca^2+^ without changing its conformation

**Fig. 6:**
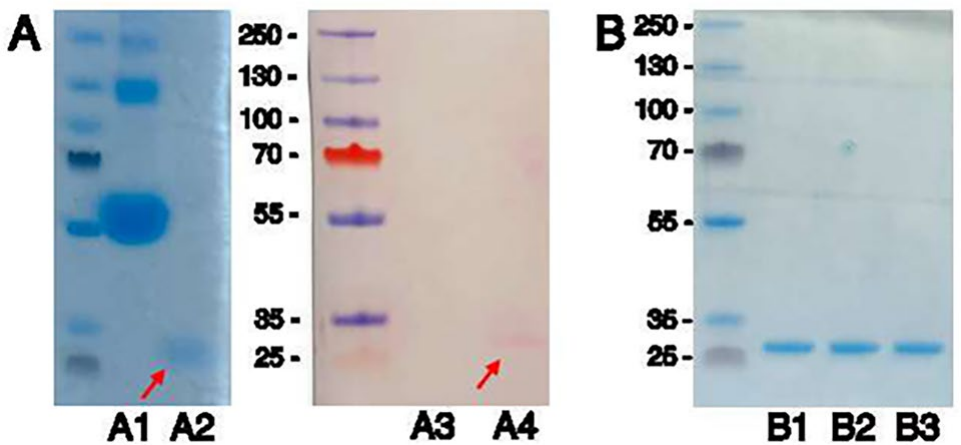
Molecular characterization of LdEP01. The mature LdEP01 protein is predicted to be 24.71 kDa with 2.21 kDa added for vector and 6x His tag (26.92 kDa). **A** Analysis of proteins stained by ruthenium red. Lanes A1 and A2: SDS-PAGE of BSA and purified LdEP01, respectively; lanes A3 and A4: the proteins were transferred to a PDVF membrane and stained with ruthenium red. The arrows indicate the band corresponding to the LdEP01 protein, stained by ruthenium red. BSA was not stained. **B** Mobility shift assay of LdEP01 purified protein following incubation with 0.5 mM EDTA (lane B1), 0.5 mM CaCl_2_ (lane B2), and 3 mM CaCl_2_ (lane B3)

To determine if LdEP01 could bind calcium, the protein was expressed in BL21 cells. A 25 kDa protein corresponding to LdEP01 expected size was stained with ruthenium red stain following native protein gel electrophoresis and transferred to a PDVF membrane (Fig. 6A). Ruthenium red binds to the calcium binding sites of these proteins and has been used to identify calcium binding proteins [29].

LdEP01 was also evaluated for conformation changes in the presence of different calcium concentrations or EDTA. No difference of mobility was identified when LdEP01 was in the presence of EDTA, 0.5 mM or 3.0 mM CaCl_2_ when subjected to SDS-PAGE electrophoresis (this experiment was repeated three times using independent protein preparations) (Fig. 5B). These results suggest that LdEP01 binds to calcium, as shown with ruthenium red stain but without a conformation change.

## Discussion

This study identified LdEP01, the first salivary effector protein in *L. delicatula*. This effector was identified from the spotted lanternfly salivary gland transcriptome and was predicted to encode a secreted protein (Fig. 1). LdEP01 modulates plant defenses by suppressing cytosolic calcium, and hypersensitive response (HR). Furthermore, we confirmed that LdEP01 is highly expressed in the salivary glands of the spotted lanternfly compared to the whole body (Fig. 2). Therefore, this gene is a candidate salivary effector. The spotted lanternfly has large salivary glands that account for 2% and 6% of the fresh body weight of adult females and males, respectively [30]. These glands secrete proteins and other molecules during feeding, allowing the insect to subvert plant defenses. A previous study has identified in the SLF salivary glands large concentrations of the plant hormones salicylic acid and abscisic acid that may modulate plant defenses [30]. However, effector proteins have not been identified in the saliva of this species.

Phloem-feeding insects secrete a plethora of effectors with diverse functions to subvert their hosts [31, 32]. Some of these effectors directly disrupt signaling and plant defenses, while others interact with host proteins [33]. Several effector proteins have been identified as modulating plant defenses [9]. For instance, ApHRC from *Acyrthosiphon pisum* suppresses both ROS and calcium levels [34]. NlugOBP11, an odorant-binding protein found in *N. lugens* plays an essential role in the survival and feeding of the pest [35]. While salivary effectors have been studied in other insect species, there is very little information on SLF.

Salivary effectors can manipulate plant defenses during the intercellular portion of the feeding process or intracellularly during probing or salivation in the sieve element [36]. Therefore, it was important to evaluate the role of the candidate effectors in both localizations. Because the sec-secretion system is conserved among eukaryotes [37], expression of the candidate gene with the signal peptide in *N. benthamiana* cells following agroinfiltration would result in its secretion outside the plant cell, mimicking the extracellular localization of a salivary effector. However, its expression without the signal peptide would not result in the secretion of the protein, mimicking the intracellular localization of an effector secreted during probing or feeding from the sieve elements [38]. Therefore, to evaluate the role of the candidate protein as a manipulator of plant defenses, it was expressed with and without the signal peptide.

LdEP01 was predicted to encode a protein with two EF-hand calcium-binding domains (Fig. 1). When threatened by attackers, plants use calcium bursts as signaling molecules to deploy different defenses [39]. In the phloem, this burst can induce callose deposition at the sieve plate and deployment of P-proteins, leading to phloem plugging [17]. EF-hand calcium-binding domains could bind calcium, disrupting plant signaling [40]. Indeed, some effector proteins can modulate calcium levels in host plants. For instance, a *N. lugens* salivary EF-hand calcium-binding effector has been shown to suppress cytosolic calcium levels in rice and bind to calcium molecules [12].

Since plants elicit different signaling cascades and defenses in response to attackers [41], we tested if the candidate effector could manipulate calcium accumulation after challenge with two different Pathogen-Associated Molecular Patterns (PAMPs). The mature form of LdEP01 disrupted the calcium accumulation after challenge with chitin (Fig. 3D), a PAMP from insects and fungi, but not flg22, a bacterial PAMP [42]. Therefore, the ability of LdEP01 to disrupt calcium accumulation only upon chitin challenge is consistent with a role of this candidate effector in manipulating defenses following insect herbivory. Salivary effectors disrupting signaling following only one type of challenge have already been reported. For instance, *Myzus persicae* effector Mp10, suppressed oxidative bursts induced by flg22 but not by chitin [10]. Following an attack, an accumulation of calcium in the cytoplasm occurs as part of the signaling process. The calcium enters the cytoplasm from the apoplast and other organelles such as the endoplasmic reticulum. Therefore, it is not surprising that only the construct without the signal peptide was able to disrupt calcium accumulation in the assays performed. The suppression of cytoplasmic calcium levels following chitin challenge suggests that LdEP01 modulates host plant calcium bursts, potentially disrupting the deployment of defenses and systemic signaling, including the disruption of defenses in the phloem, such as callose deposition and phloem plugging, allowing the pest to feed efficiently [40]. The fact that LdEP01 suppressed calcium accumulation in response to chitin but not flg22 might be related to the activation of different signaling cascades, since this molecules are recognized by different receptors and activate different signaling cascades. Alternatively, it could be linked to differences in calcium accumulation by each elicitor: flg22 induced a stronger response. Similar observations were reported when analyzing *Pseudomonas syringae* effectors [43].

Because LdEP01 was able to suppress cytoplasmic calcium, we evaluated if it could bind to calcium and change its conformation in the presence of calcium. The purified LdEP01 protein was stained by ruthenium red, confirming that this protein contains calcium-binding sites (Fig. 5A). The ruthenium red stain has been previously used to identify calcium-binding saliva proteins [29, 40]. After confirming that LdEP01 could bind calcium, we tested whether the presence of calcium induced a change in protein conformation using a mobility shift assay. However, we did not observe a mobility change when the protein was incubated with calcium or with EDTA, a calcium chelator (Fig. 5B). Staining by ruthenium red without a conformational change was also found in another EF-hand calcium-binding salivary protein, LtRIN in *Aedes aegypti* [44]. On the other hand, a conformational change in the presence of calcium was noted for the *N. lugens* EF-hand effector NlSEF1 [12]. It is important to note that the conformation assays in this study were performed by evaluating different calcium concentrations but lacked a positive control. While several salivary effectors encoding EF-hand calcium-binding domains have been identified, this is the first candidate also encoding an FKBP-type domain. These domains are found in peptidylprolyl isomerases, which are chaperone proteins that accelerate protein folding by catalyzing the cis-trans isomerization of proline imidic peptide bonds in oligopeptides [45].

In *N. benthamiana*, a cytosolic Ca^2+^ concentration threshold is required for ROS production [46]. Therefore, we hypothesized that LdEP01 could also reduce ROS signaling, particularly following chitin perception. Contrary to the expectation, our assays revealed that LdEP01 expression resulted in higher ROS burst upon chitin challenge when the protein was expressed with or without the signal peptide, compared to the empty vector (Fig. 4C and D), while only the expression of the protein without the signal peptide resulted in higher ROS accumulation upon flg22 challenge (Fig. 4B). The increased ROS accumulation upon challenge in leaves expressing the candidate effector indicates that LdEP01 does not suppress ROS signaling, which is counterintuitive when compared to typical responses during bacterial infection. Our results suggest that LdEP01 might enable ROS accumulation at lower Ca^2+^ levels or that it may uncouple the canonical calcium–ROS signaling relationship, potentially allowing ROS accumulation even under conditions of reduced cytosolic Ca²⁺. The mechanism behind these results remains unclear and could be linked to the role of the FKBP-type domain present in LdEP01. These results highlight the complexity and context-dependence of plant immune signaling. plant immune response.

Furthermore, ROS negatively regulates the calcium channels responsible for the Ca^2+^ influx [46]. Therefore, it is plausible that LdEP01 disrupts calcium accumulation both directly, by binding Ca^2+^, and indirectly, by inducing high ROS accumulation leading to the negative regulation of Ca^2+^ channels. However, this latter effect alone is not sufficient to reduce calcium accumulation because ROS levels increased in response to both flg22 and chitin, but calcium signaling was only affected in response to chitin.

Several examples of salivary proteins eliciting plant defenses have been reported; in many cases, these proteins induce defenses and decrease insect performance. For example, *M. persicae* effector CathB3 induces ROS accumulation in tobacco [47]. But insects secrete an abundance of salivary proteins that also suppress ROS, such as *M. persicae* Mp10 [10], potentially helping suppress plant defenses. Furthermore, some effectors can both, be recognized by the plant alert system, eliciting defenses, and subsequently, disrupt downstream signaling events. ROS are at the same time signaling and defense molecules; therefore, the increased accumulation of ROS upon challenge in leaves expressing LdEP01 might reflect that defenses were induced following the recognition of the effector. To evaluate if LdEP01 could disrupt plant defenses downstream signaling events, we tested if this effector could disrupt Prf^D1416V^-induced cell death, a defense response that occurs downstream signaling. Prf^D1416V^ is an autoactive form in *N. benthamiana* of the Prf R protein causing effector-independent programmed cell death (22). Both LdEP01 with and without the signal peptide disrupted Prf^D1416V^-induced cell death (Fig. 5A and B). These results suggest that while this protein might suppress calcium bursts but not ROS signaling, it is able to disrupt immune responses. Plants and their enemies are engaged in an evolutionary arms race; it is possible that the same protein can elicit defenses that are then suppressed downstream. AvrPtoB is an example of an effector able to induce cell death but also suppress programmed cell death in plants [48]. Cell death is a response of plants upon attack and phloem-feeding hemipterans need to disrupt this defense to feed efficiently. While here we used Prf^D1416V^-induced cell death as a tool to determine if the candidate effector could disrupt plant immune responses, effectors that disrupt effector-triggered immunity cell death can play a significant role in allowing insects to feed. Indeed, the *Vat* gene induces programmed cell death upon aphid feeding, conferring melon plant resistance to *Aphis gossypii* [49].

All the assays presented here were conducted in *N. benthamiana*, but the second messengers of the signaling cascades are the same among plant species. Therefore, a salivary effector that binds calcium can be involved in suppressing plant defenses in a wide range of hosts. Experiments evaluating SLF feeding and performance when this effector is silenced are needed to assess the insect’s reliance on this protein to manipulate plant defenses.

**Fig. 7:**
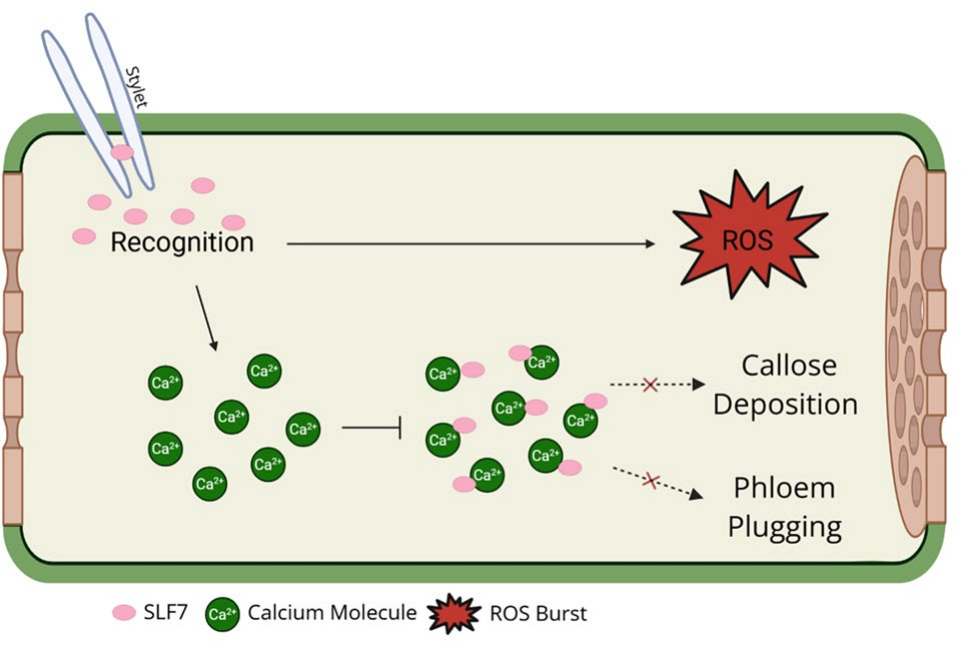
Proposed model for LdEP01 showing the proteins’ ability to manipulate plant defenses in the phloem. LdEP01 binds and suppresses calcium levels but it does not suppress oxidative bursts. A consequence of reducing calcium burst might be reducing callose deposition and phloem plugging. The model was created in BioRender by Smith, A. (2025) https://BioRender.com/8rp5nl2

Based on the results of this study, LdEP01 is a predicted secreted effector protein of the spotted lanternfly that is highly expressed in the salivary glands. LdEP01 was able to bind and suppress cytosolic Ca^2+^ levels but showed no conformational change in the presence of Ca^2+^. LdEP01 was also able to suppress induced cell death (Fig. 7). Because plant species use the same signaling events and second messengers in signaling cascades, this effector is a candidate to facilitate feeding by the spotted lanternfly in any host it might be secreted. These results suggest that this candidate effector of the spotted lanternfly has a significant role in modifying plant defenses, allowing this pest to feed efficiently.

## Supplementary Information

Below is the link to the electronic supplementary material.

Supplementary material 1 (DOCX 65.6 kb)

Supplementary material 2 (TIFF 303.0 kb)

Supplementary material 3 (TIF 87.5 kb)

Supplementary material 4 (TIF 134.9 kb)

Supplementary material 5 (TIFF 971.5 kb)

## Data Availability

All data were included in the manuscript.
