# Supplementary figures and images for: LdEP01, the first characterized Lycorma delicatula salivary effector protein modulates plant defenses
Source: Mol Biol Rep. 2025 Dec 11;53(1):191. doi: 10.1007/s11033-025-11288-3 (PMC12698798; doi:10.1007/s11033-025-11288-3)

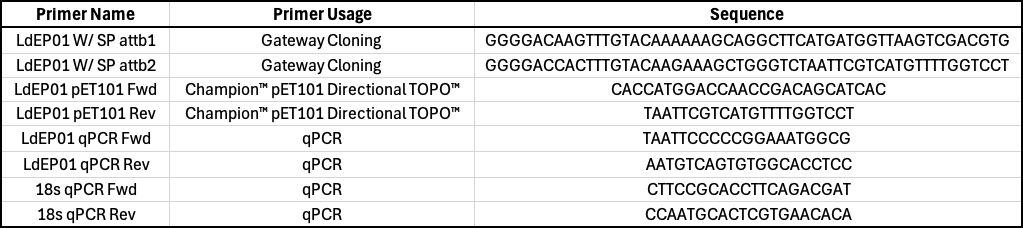

Supplement: Supplementary file 1 — Supplementary material 1 (DOCX 65.6 kb) [file 11033_2025_11288_MOESM1_ESM.docx]

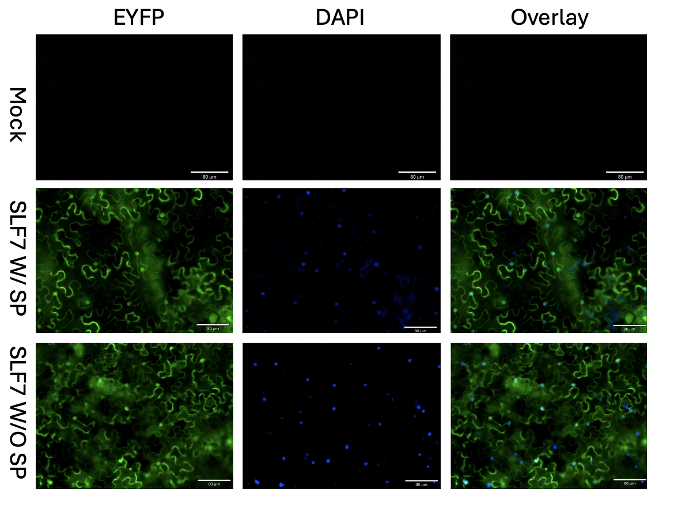

Supplement: Supplementary file 2 — Supplementary material 2 (TIFF 303.0 kb) [file 11033_2025_11288_MOESM2_ESM.tiff]

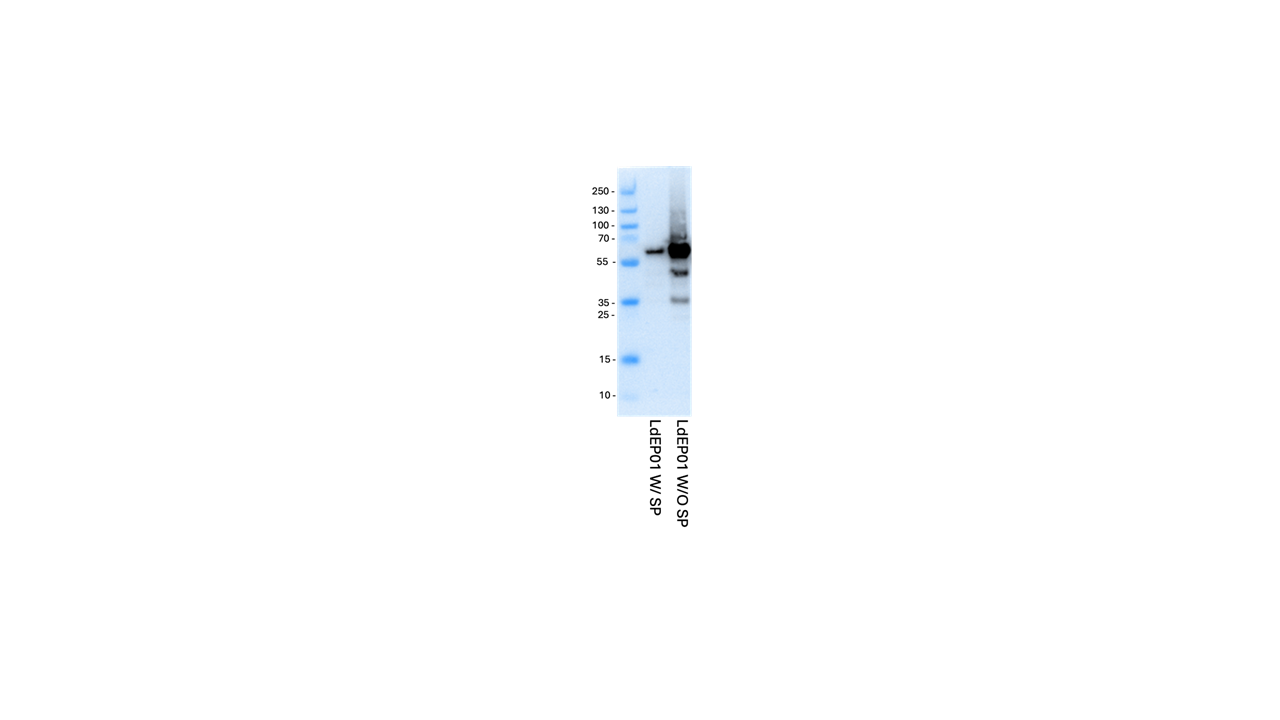

Supplement: Supplementary file 3 — Supplementary material 3 (TIF 87.5 kb) [file 11033_2025_11288_MOESM3_ESM.tif]

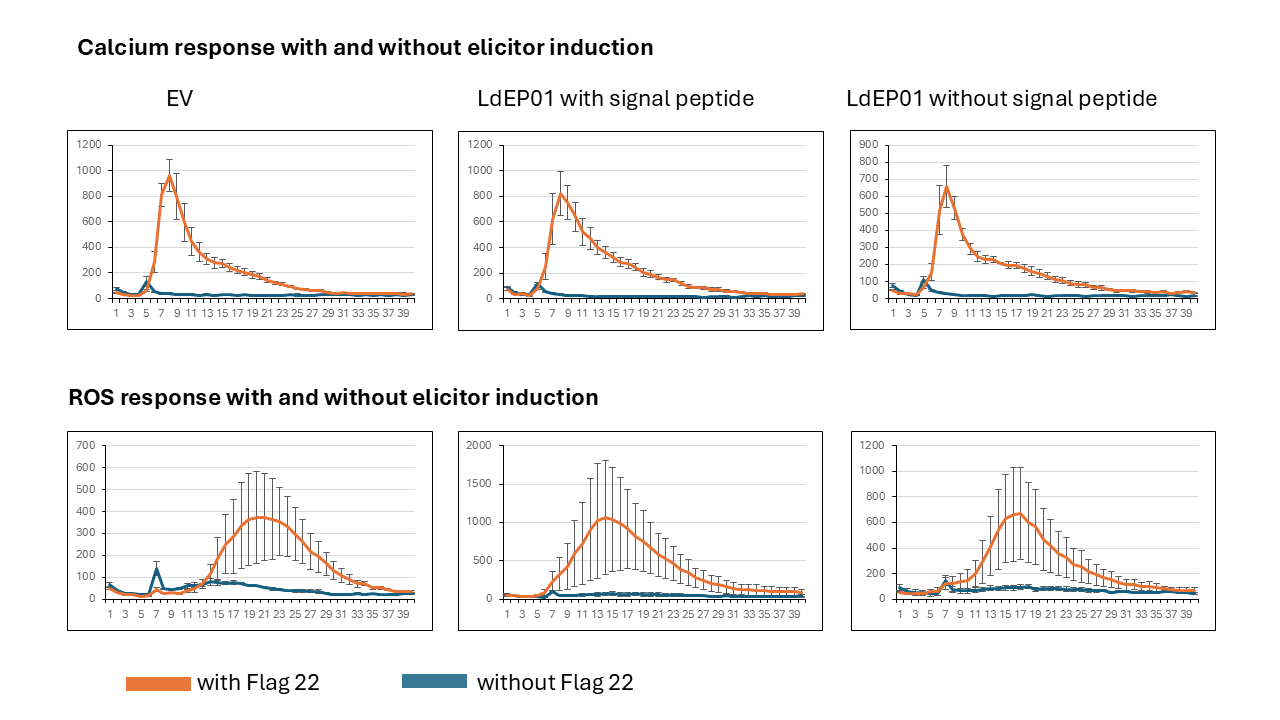

Supplement: Supplementary file 4 — Supplementary material 4 (TIF 134.9 kb) [file 11033_2025_11288_MOESM4_ESM.tif]

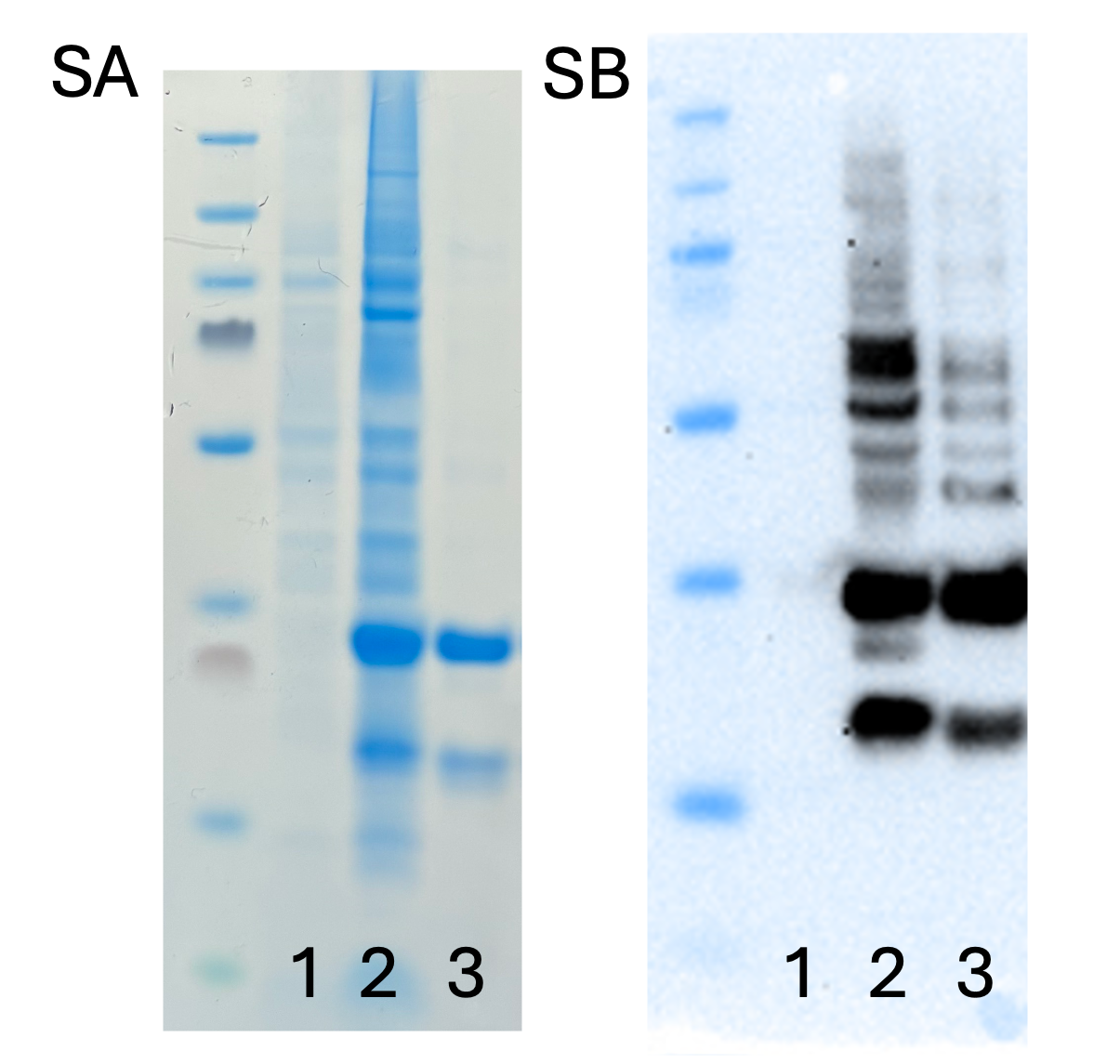

Supplement: Supplementary file 5 — Supplementary material 5 (TIFF 971.5 kb) [file 11033_2025_11288_MOESM5_ESM.tiff]
